# Systems pharmacology-based approach for dissecting the mechanisms of pyrazine components in Maotai liquor

**DOI:** 10.1042/BSR20191864

**Published:** 2019-10-11

**Authors:** Wei Zhou, Ziyi Chen, Guohao Zhang, Zhigang Liu

**Affiliations:** 1 The Research Center of Allergy & Immunology, Shenzhen University School of Medicine, Shenzhen 518060, China; 2 Department of Respirology & Allergy, Third Affiliated Hospital of Shenzhen University, Shenzhen 518020, China

**Keywords:** Diseases, Maotai liquor, Pharmacological mechanisms, Pyrazine components, Systems pharmacology

## Abstract

Maotai liquor is a typical representative of sauce aroma-style flavor liquors and has been considered to be a precious cultural heritage of the oriental spirit culture. Aroma components are largely responsible for the characteristic aroma of liquor. Pyrazine compound is one of the most important categories of aroma components that affect the flavor of Maotai liquor. However, limited information is available regarding the systemic analysis of pyrazine compounds, especially the pharmacological effects of bioactive pyrazine components. Therefore, in the current study, a systemic analysis approach was provided by integrating absorption, distribution, metabolism, and excretion (ADME) screening, target identification, pharmacological evaluation and pathway analysis to explore the pharmacological mechanism of pyrazine compounds in Maotai liquor. As a result, 17 pyrazine components with adequate pharmacokinetic properties were filtered out using ADME models. Thirty eight potential targets of these active compounds were identified through target prediction. The pharmacological evaluation was proposed to uncover the pharmacological effect of pyrazine compounds in Maotai liquor from the holistic perspective. Finally, the pharmacological effects of the pathways perturbed by potential targets were interpreted based on the pathway analysis. Our study lays the foundation for formulating a comprehensive understanding of the pyrazine compounds in Maotai liquor, which would contribute to the development of Chinese liquor.

## Introduction

Chinese liquor is a traditional fermented alcoholic drink that plays a particular role in Chinese traditional culture and people’s daily lives owing to its historical and cultural factors. According to the aroma characteristics, they can generally be classified into the following categories: soy sauce aroma type, strong aroma type, light aroma type, sweet and honey type, and miscellaneous aroma type. Among them, the soy sauce aroma type of Chinese liquor has been recognized as highest quality and requires the most intricate procedures of production. As the most famous one, Maotai liquor is belonged to this family that possesses a unique flavor of highly complex-flavored, sweet, and refreshing [1]. It has a long history of distillation and some outstanding qualities, considering as a symbolic drink in China ranked alongside whisky in Scotland, and brandy in France [2]. The production of Maotai liquor is mainly based on special fermentation technology using a mixture of milled wheat and a complex microbial community called Maotai Daqu. During the process of Daqu fermentation, many microorganisms and abundant enzymes originating from the microbes can produce various aroma components, which are the determinative factors for the liquor’s flavoring.

Pyrazine is one of the most aroma active compounds in soy sauce aroma type of Chinese liquor. It is considered to be an important nitrogen-containing heterocyclic molecule that is directly related to food flavoring [3]. Although analytical chemists have conducted to identify the pyrazine compounds from Maotai liquor [2,4], the systemic analysis concerning the pharmacological effect of pyrazine compounds in Maotai is limited. In particular, the relationships between the bioactive pyrazine molecules and their related target proteins are still unclear.

Even conventional experimental approaches have made some progresses in extraction, isolation and identification of the compounds from Maotai liquor, they are still time-consuming, laborious and high-costy. Because of the structural diversity of compounds and the complicated synergistic mechanisms of multiple components, it is extremely difficult to analyze the complex interactions between active compounds and their relevant targets at a systems level. Alternatively, the emergence of systems pharmacology method provides a new way to investigate such complex relationships and is capable of avoiding limitations brought about by conventional experiments. Systems pharmacology as an emerging field provides a new method to explore the mechanism of action of multi-components and interrelationships of complex networks. In previous studies, system pharmacology framework has been successfully used to identify the active compounds, target proteins and their interactions of food, to clarify the mechanisms of functional food for disease therapy, to interpret the Chinese tradition medicine theory, and to reveal the pharmacological synergy mechanisms of herb pairs [5–7].

Therefore, in the current study, a systematic analysis method was employed to reveal the active compounds, related targets and pharmacological mechanism of pyrazine compounds in Maotai liquor by combining *in silico* absorption, distribution, metabolism, and excretion (ADME) evaluation, targets identification, pharmacological evaluation, and pathway analysis. We firstly screened the active compounds in Maotai liquor by *in silico* oral bioavailability (OB) model. Then the potential targets of obtained active components were identified through the multiple target fishing method. Subsequently, the constructed compounds-targets-diseases (C-T-D) network and the cellular experiment of the key active compounds were involved to clarify the mechanism of action by the pharmacological evaluation. Furthermore, the pharmacological effects of the pathways perturbed by potential targets were interpreted based on the pathway analysis. Our study provides not only a novel perspective for the systems-level understanding of the pyrazine compounds in liquor, but also a rational way for promoting the in-depth research of complex components in Chinese liquors.

## Materials and methods

**Figure 1:**
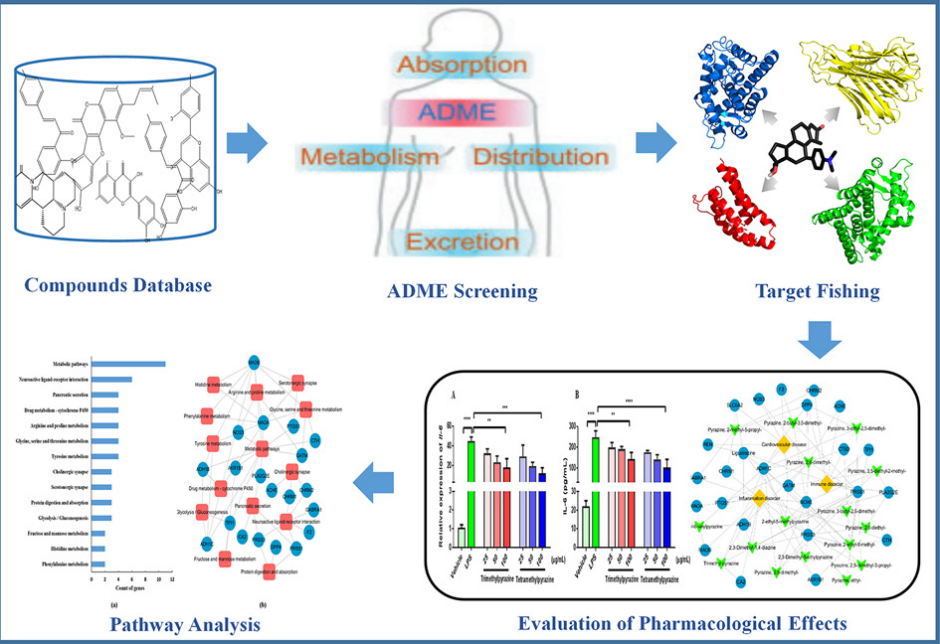
The framework of systems pharmacology approach

In order to clarify the pharmacological effects of pyrazine compounds in Maotai liquor, an integrated systems pharmacology framework including ADME evaluation, targets fishing, network pharmacology technology, pathway analysis, and experimental verification was developed. Figure 1 shows the flowchart of the systems pharmacology approach.

### Dataset construction

Nineteen compounds were obtained from the references published in the past years. The structures of these components were gathered from The PubChem project (https://pubchem.ncbi.nlm.nih.gov/) and were saved in mol2 format for further analysis.

### Active compounds screening

ADME has been considered to be an essential procedure to identify candidate molecules with potential biological effect in the early stages of the drug discovery process [8]. OB, as one of the most crucial ADME parameters of orally administered drugs, determines the rate and percentage of an oral dose for a compound that is absorbed into systemic circulation and exerts pharmacological effects. In the current work, an *in silico* model OB was involved to calculate the OB values of all pyrazine compounds in Maotai liquor, which was established using 805 diverse drugs or drug-like molecules through consideration of the action of P-glycoprotein and Cytochrome P450s in metabolism and information transport [9]. Ultimately, the molecules with OB ≥ 29% were filtered as the candidate compounds for further analysis since the average oral absorption and bioavailability of a drug is 30% with 10–60% variability according to the past reports [10,11]. Furthermore, two careful considerations were also employed for establishment of the threshold determination. First, extract information from Maotai liquor as much as possible with the least number of pyrazine compounds. Second, the obtained model can be rationally explained by the published pharmacological data.

### Targets identification

A robust prediction model based on two powerful methods named support vector machine (SVM) and random forest (RF) was employed to identify the targets of bioactive pyrazine compounds in Maotai liquor [12]. This model was constructed by integrating the chemical, genomic and pharmacologic information for compound targeting on a large scale and exhibited good performance of the prediction for compound-target interactions, with the concordance of 85.83%, the sensitivity of 79.62%, and the specificity of 92.76%, respectively. Generally, in SVM and RF models, setting the output expectation score to at least 0.5 shows a good affinity prediction of the compounds to a target protein [13]. Therefore, in current work, the proteins with the score values obtained by SVM and RF methods larger than 0.7 are chosen as the potential targets of active pyrazine compounds for improving the accuracy of prediction. All the obtained targets were sent to the therapeutic target database to extract their related diseases.

### Evaluation of pharmacological effects

#### Network pharmacological analysis

To investigate the pharmacological effect of active pyrazine compounds in Maotai liquor at a network level, two kinds of visualized networks were established: (1) the C-T-D network was constructed by connecting the active compounds, potential targets, and their related diseases. (2) The target-pathway (T-P) network was produced to explore the complex relationship between the targets and pathways. In these network, nodes indicate compounds, targets, diseases or pathways, and edges indicate C-T-D or T-P interactions. The networks were generated by a popular bioinformatics package Cytoscape 3.2.1 for biological data integration and analysis [14].

#### Anti-inflammatory effects of screened key active compounds

##### Cell culture and treatment

The murine macrophage cell line, RAW 264.7, was obtained from American Type Tissue Culture Collection (ATCC) and maintained in DMEM containing 10% FBS and 1% penicillin/streptomycin at 37 °C and 5% CO_2_ in a humidified atmosphere. Confluent cells in six-well plate were pre-treated with trimethylpyrazine and tetramethylpyrazine (Aladdin) for 2 h and then stimulated by lipopolysaccharides (LPS) (sigma, 1 μg/ml). DMSO was used as vehicle control. The chemicals and LPS were maintained in medium until sample collection. After 24 h, the supernatant was collected for ELISA assay and cells were harvested for RNA extraction.

##### Cell viability assay

Cell viability was evaluated by Cell counting KIT-8 (CCK-8) assay according to manufacture’s instructions. Cells were seeded in 96-well plates at a density of 1×10^4^ cells/100 µl/well and cultured for 24 h with different concentrations of trimethylpyrazine or tetramethylpyrazine. At the end point, 10 µl CCK-8 reagent was added and incubated for 3 h. Absorbance rate was measured at 450 nm.

##### Real-time PCR, ELISA, and statistics

Total RNA was extracted from drug-treated cells by TRIzol reagent (Invitrogen, U.S.A.). One microgram RNA with OD 260/280 <2.0 and >1.8 was used for reverse transcription by PrimeScript RT Reagent Kit (Takara). The information of primers was as follows: *Gapdh* (forward) 5’-AACGACCCCTTCATTGAC-3’, *Gapdh* (reverse) 5’-TCCACGACATACTCAGCAC-3’, *Interleukin-6* (*IL-6*, forward) 5’-CCAAGAGGTGAGTGCTTCCC-3’, *Il-6* (reverse) 5’-CTGTTGTTCAGACTCTCTCCCT-3’. The concentration of IL-6 in culture medium was measured by ELISA kit (EK-Bio) according to manufacturer’s instructions.

All results were repeated three times and analyzed by one-way ANOVA, followed by Dunnett’s test. *P*<0.05 was considered as significant difference.

## Results and discussion

### Active compounds filtering

To determine pharmaceutically active components from pyrazine compounds in Maotai liquor, an *in silico* model OB was involved in the present study. The components with the OB ≥ 29% were chosen as candidate compounds for further analysis. As a result, 17 potential active compounds with OB ≥ 29% were obtained, including 2-methyl-6-vinylpyrazine (OB = 54.49%), pyrazine, 2,6-diethyl- (OB = 52.41%), trimethyl-pyrazine (OB = 32.47%), and so on. The detailed information of screened active compounds is shown in Table 1.

**Table 1:** The information of 17 candidate pyrazine compounds in Maotai liquor

Compounds	CAS	OB	Structure
2-methyl-6-vinylpyrazine	13925-09-2	54.49	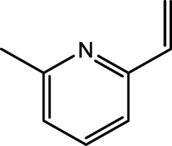
2-ethyl-5-methylpyrazine	13360-64-0	44.19	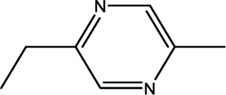
Trimethylpyrazine	14667-55-1	32.47	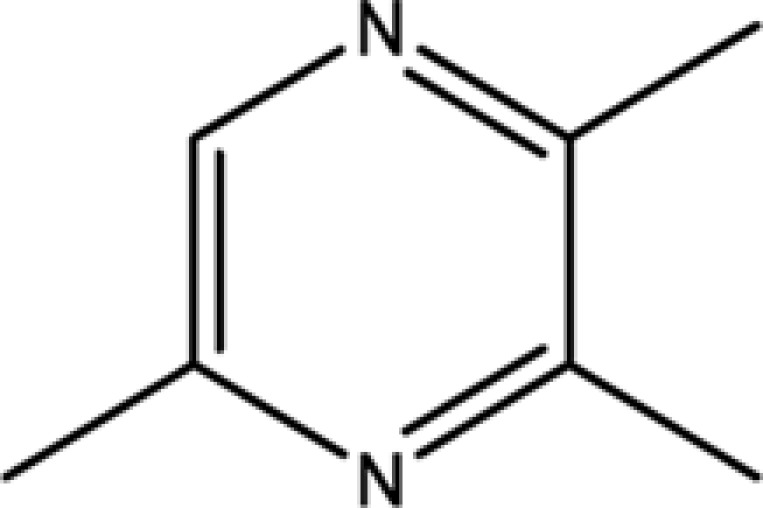
Pyrazine, ethyl-	13925-00-3	35.29	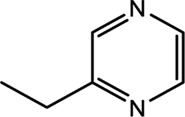
Pyrazine, 3-ethyl-2,5-dimethyl-	13360-65-1	33.23	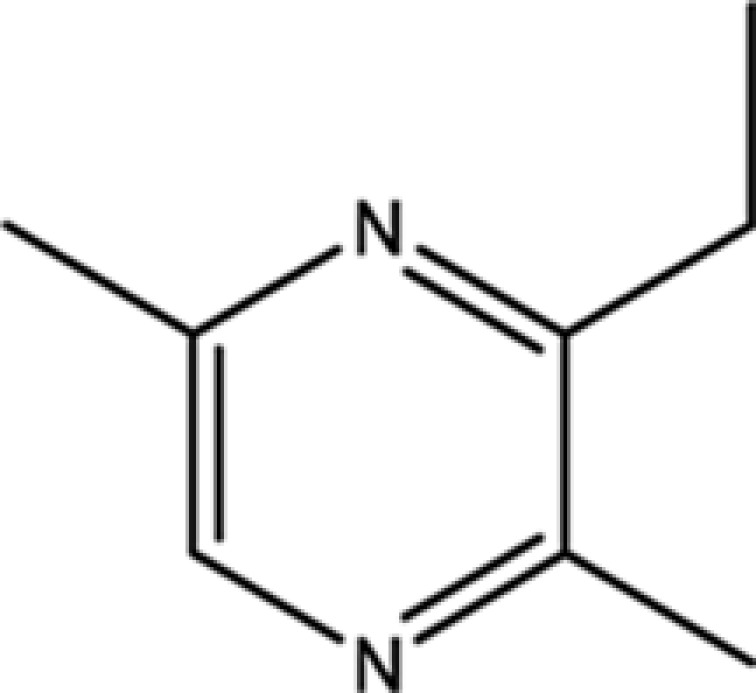
Pyrazine,3-butyl-2,5-dimethyl-	40790-29-2	29.57	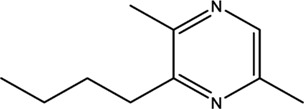
Pyrazine,3,5-diethyl-2-methyl-	18138-05-1	31.22	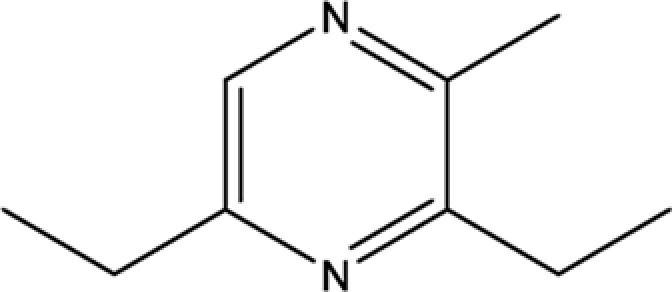
Pyrazine,2-methyl-5-propyl-	29461-03-8	40.37	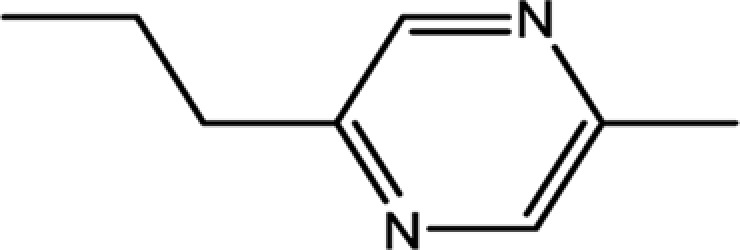
Tetramethylpyrazine	1124-11-4	29.22	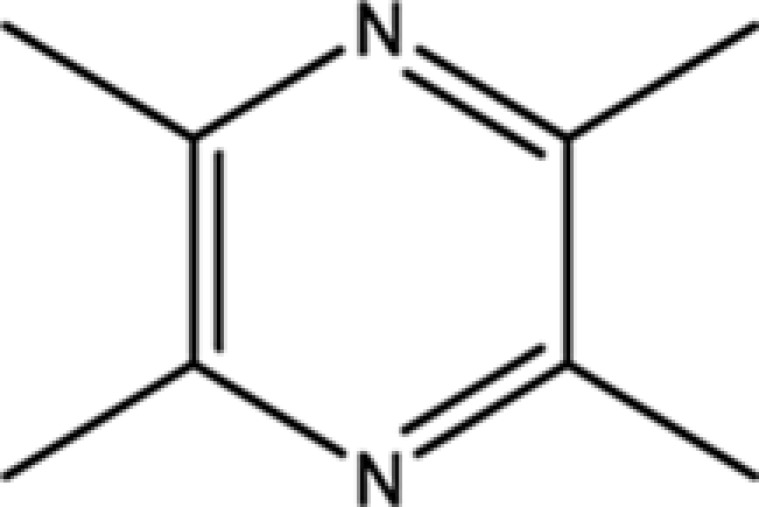
Pyrazine,2-ethyl-6-methyl-	13925-03-6	37.44	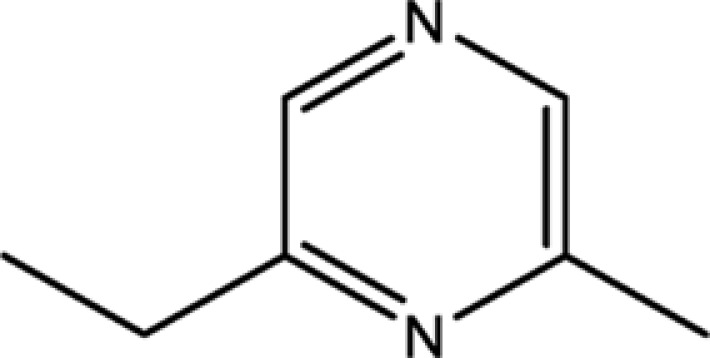
Pyrazine, 2-butyl-3,5-dimethyl-	50888-63-6	35.053	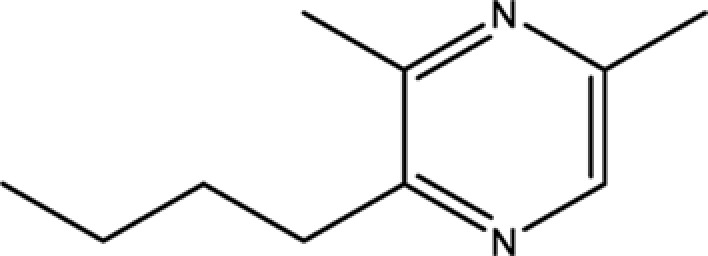
Pyrazine, 2,6-dimethyl-	108-50-9	35.27	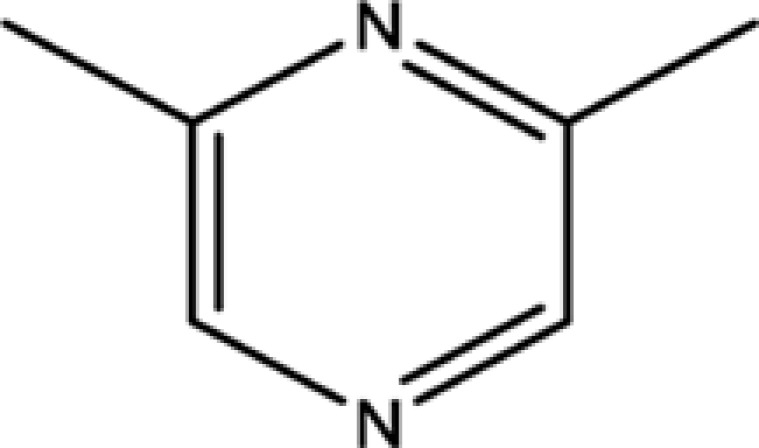
Pyrazine, 2,6-diethyl-	13067-27-1	52.41	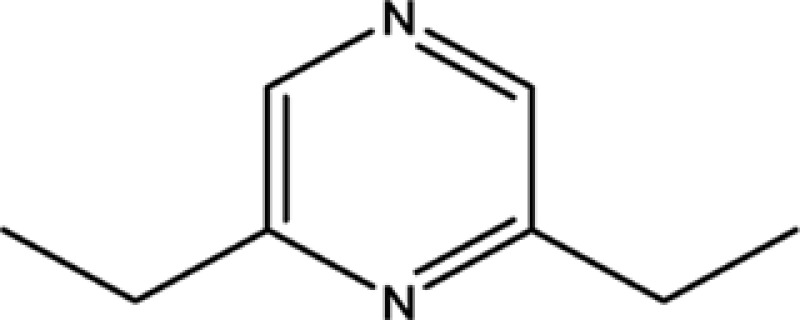
Pyrazine, 2,5-dimethyl-3-propyl-	18433-97-1	39.75	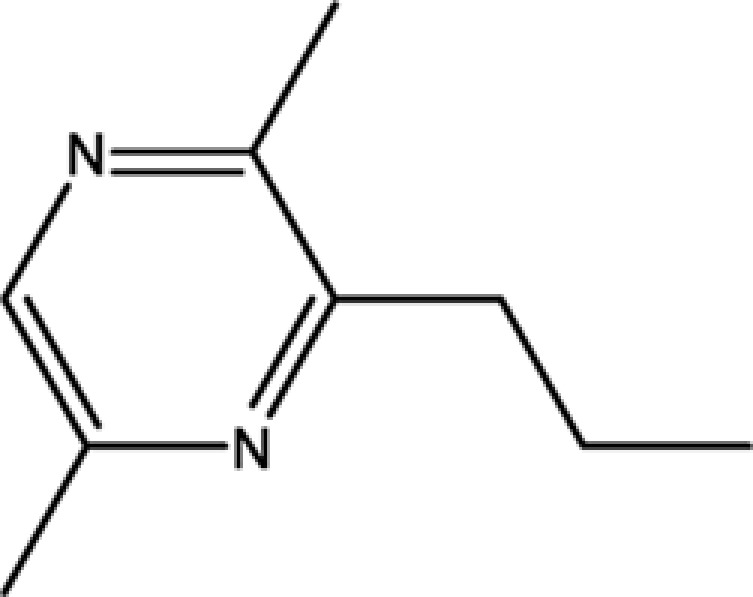
Pyrazine, 2,5-dimethyl-	123-32-0	33.80	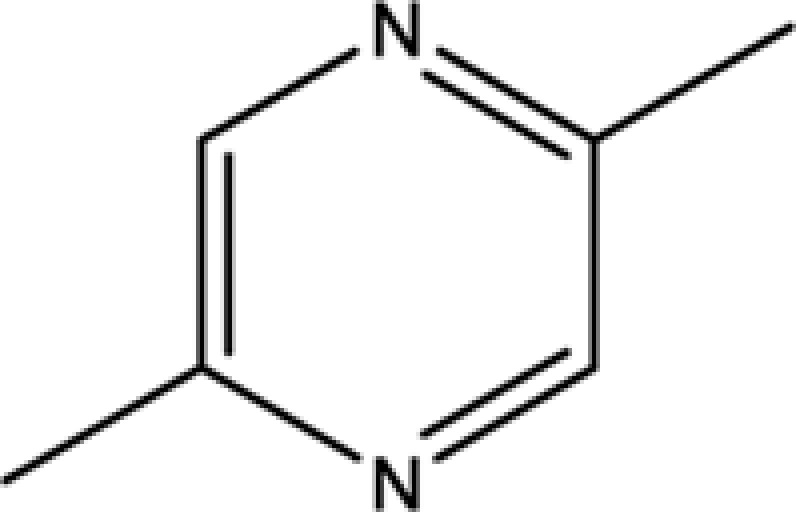
2,3-Dimethyl-5-ethylpyrazine	15707-34-3	38.29	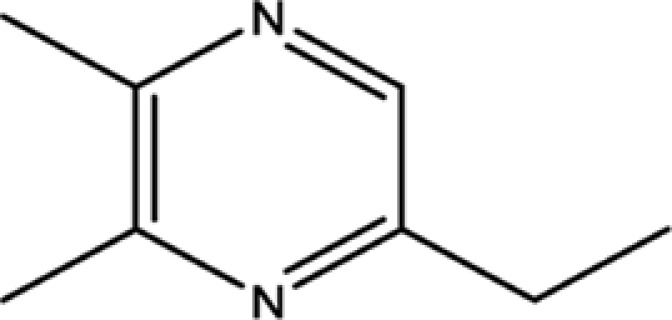
2,3-Dimethyl-1,4-diazine	5910-89-4	30.00	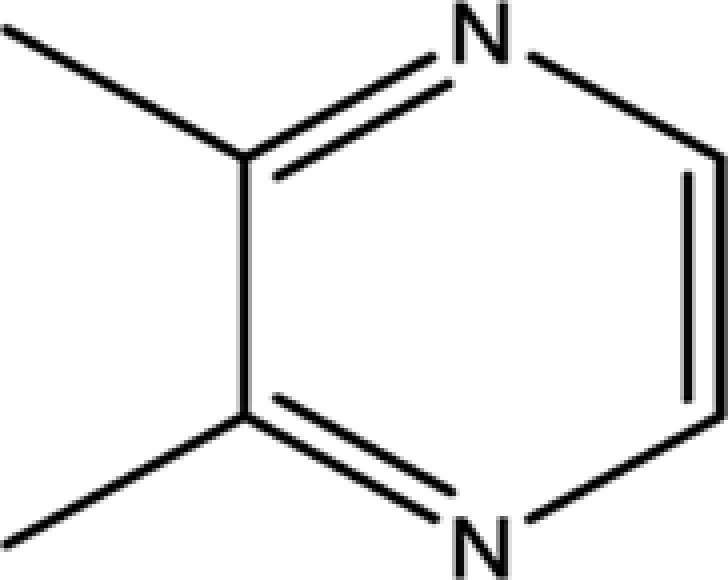

### Target identification

To determine the underlying molecular mechanism of pyrazine compounds in Maotai liquor, a robust model DTpre was involved to identify the targets of the candidate bioactive compounds. As a result, 25 potential targets were obtained for 17 active pyrazine compounds with 75 interactions (Supplementary Table S1). The results showed that the majority of active compounds can interact with more than one target, implying multiple pharmacological effects of the candidate pyrazine compounds. For instance, compound pyrazine, 2,6-dimethyl- targets on 11 proteins including dipeptidyl peptidase 4 (DPP4) and trypsin-3 (PRSS3), while Pyrazine, 2-ethyl-6-methyl- has interactions with 9 target proteins, such as cholinesterase (BCHE) and aldose reductase (AKR1B1).

### Evaluation of pharmacological effects

In order to systematically explore the pharmacological effects of active pyrazine compounds in Maotai liquor, the C-T-D network was employed to disclose the complex relationships between compounds, targets, and their related diseases. In addition, the anti-inflammatory effects of key active compounds is carried out by *in vitro* experiment to further validate the reasonability of our approach.

### Network pharmacological analysis

**Figure 2:**
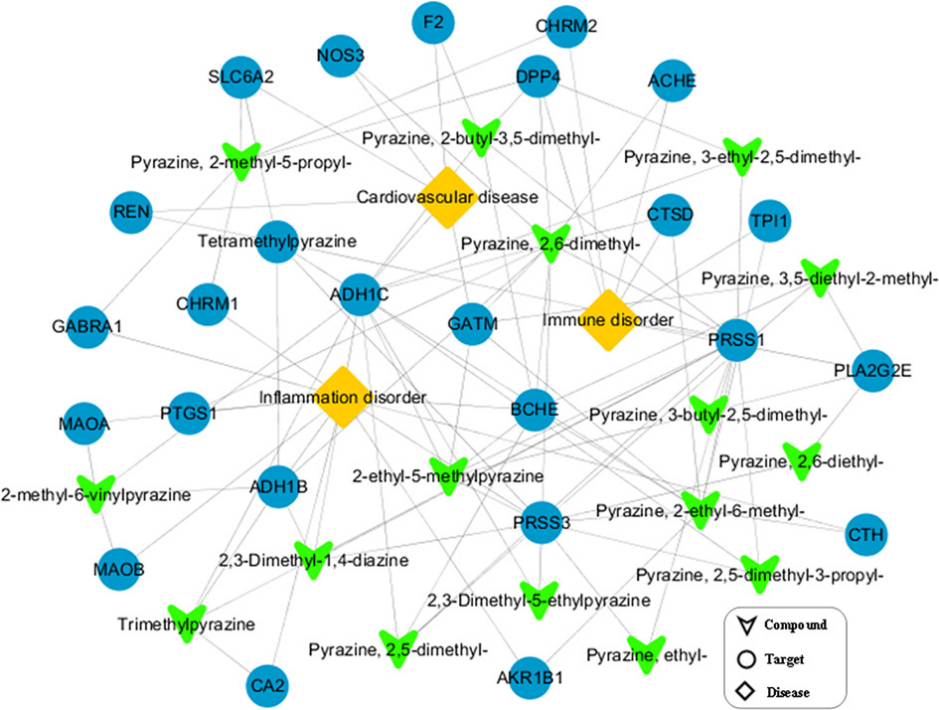
C-T-D network of active pyrazine compounds in Maotai liquor

To highlight the relationship between the targets and disease, all the candidate targets were sent to the therapeutic target database to obtain their relevant diseases. Finally, a C-T-D network that connected 17 active compounds, 25 potential protein targets, and 3 related diseases was generated to further interpret the underlying mechanisms of pyrazine compounds in Maotai liquor (Figure 2, Supplementary Table S1). Interestingly, most of these targets are likely associated with the pathogenesis of cardiovascular disease, immune, and inflammation disorders.

Cardiovascular disease as the leading cause of death in the world has attracted unprecedented attentions from medical researchers [15]. The C-T-D network revealed that 14 pyrazine compounds had satisfactory pharmacological effects on cardiovascular disease with 6 related targets (Figure 2, Supplementary Table S1). For example, target protein alcohol dehydrogenase 1C (ADH1C) hit by the highest number [12] of active compounds (tetramethylpyrazine, trimethylpyrazine, pyrazine, 2,5-dimethyl-, and so on) has been revealed to modify the influence of alcohol intake on the risk of cardiovascular disease [16]. Two active compounds (tetramethylpyrazine and pyrazine, 2-methyl-5-propyl-) have interactions with target protein sodium-dependent noradrenaline transporter (SLC6A2), which is a significant regulator of adrenergic neurotransmitters and plays a role in lowering blood pressure and regulating the functions of the cardiovascular system [17]. These results revealed that the active compounds interacting with related targets may contribute to decrease the risk of cardiovascular disease.

Immune system is essential for our survival and protects our body against invaders, such as viruses, bacteria, and other potentially damaging foreign bodies. According to the C-T-D network (Figure 2, Supplementary Table S1), seven proteins have been identified to correlate with immune disorders, such as trypsin-1 (PRSS1), acetylcholinesterase (ACHE) amd cathepsin D (CTSD). For instance, PRSS1 hit by 12 active compounds (tetramethylpyrazine, trimethylpyrazine, 2,3-dimethyl-1,4-diazine, and so on) is involved in the regulation immune response via lipopolysaccharide-stimulated toll-like receptor 4 signaling [18]. Four compounds (pyrazine, 2,6-dimethyl-, pyrazine, 2-butyl-3,5-dimethyl-, pyrazine, 2-methyl-5-propyl-, and pyrazine, 3-ethyl-2,5-dimethyl-) were against the DPP4, a multifunctional protein that is both involved in the modulating T-cell function and is responsible for the pathological process of various autoimmune diseases [19]. The results suggested that the regulation of these targets may be beneficial for improving the function of the human immune system.

Inflammation is a necessary part of the complex protective response of body to harmful stimuli that has been considered to be associated with the pathological processes of many diseases. In our C-T-D network (Figure 2, Supplementary Table S1), 11 inflammation-related targets related to 15 active compounds were identified. For instance, the protein BCHE as an enzyme targeted by six active compounds (pyrazine, 2,5-dimethyl-, pyrazine, 2,6-dimethyl-, pyrazine, 2-butyl-3,5-dimethyl-, pyrazine, 2-ethyl-6-methyl-, pyrazine, 3,5-diethyl-2-methyl-, and 2-ethyl-5-methylpyrazine) has a potential role in promoting the systemic inflammatory response through interacting with inflammatory markers like IL-6 and C-reactive protein [20]. Alcohol dehydrogenase 1B (ADH1B) targeted by four compounds (tetramethylpyrazine, 2,3-dimethyl-1,4-diazine, pyrazine, 2,6-dimethyl-, trimethylpyrazine, and 2-methyl-6-vinylpyrazine) has been reported to be involved in the pathogenesis of inflammation, the abrogation of which will activate the tumor-promoting inflammation in colorectal cancer [21]. These findings may contribute to demonstrate the anti-inflammation effects of the candidate pyrazine compounds.

### Anti-inflammatory effects of screened key active compounds

The anti-inflammatory effects of screened key chemicals (trimethylpyrazine and tetramethylpyrazine) were detected in LPS-induced macrophage cell model to evaluate the reliability of our strategy.

**Figure 3:**
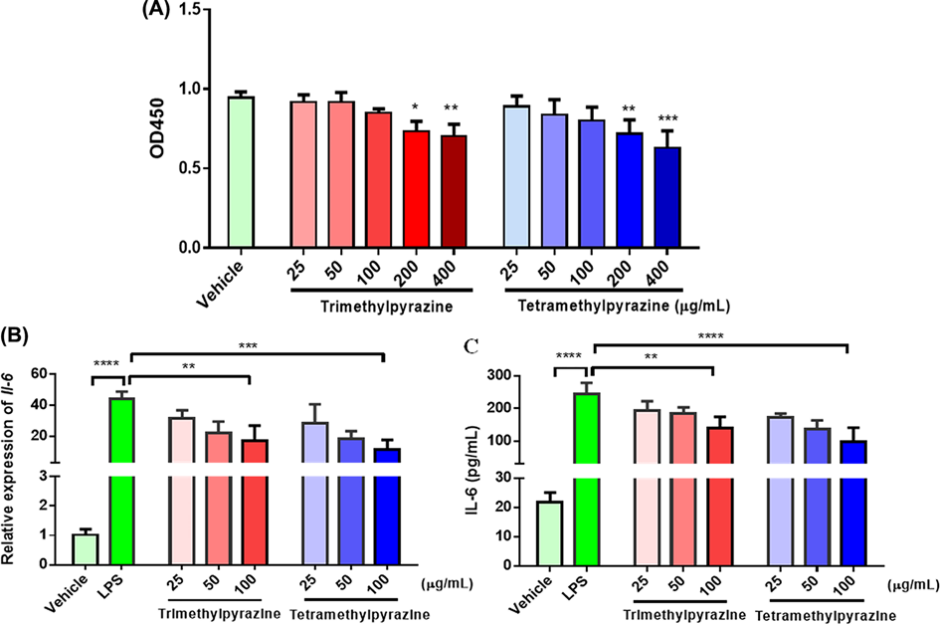
Detection of cell viability and the expression of inflammatory cytokine IL-6 in RAW 264.7 cells after chemical treatment (**A**) The cell viability was determined by the values of OD450. Various doses of trimethylpyrazine and tetramethylpyrazine (25–400 μg/ml) were used to treat cells for 24 h. N = 3 (**B**) The transcriptional level of *Il-6* was detected in cells pretreated with trimethylpyrazine (25, 50, and 100 μg/ml) and tetramethylpyrazine (25, 50, and 100 μg/ml). DMSO was used as vehicle control. N = 3. (**C**) The paracrined level of IL-6 in supernatant was detected by ELISA kit. The concentration of labeled protein was calculated according to the standard curve. N = 3. Data are shown as mean ± SEM, **P <0.01, ***P <0.001, ****P <0.0001.

In Figure 3A, we firstly explored the cytotoxicity of the two chemicals by CCK-8 assay. The results showed that doses of 200 and 400 µg/ml caused significant cytotoxicity indicating doses under 100µg/ml were safe during the incubation of 24 h. Thus concentrations of 25, 50, 100 µg/ml were used in the following experiments. The expressions of IL-6 at transcriptional and protein level were measured by real-time PCR and ELISA (Figure 3B,C). LPS stimulated the inflammatory response indicated by 40 times (*P*<0.0001) increase of endogenous mRNA and 21 times increase of supernatant secreted protein compared with vehicle control. Macrophage cells pretreated with both chemicals showed abated inflammatory response after chemical treatment in a dose-dependent manner. Trimethylpyrazine and tetramethylpyrazine at the concentration of 100μg/ml suppressed both transcriptional and protein level of IL-6 to approximately 57% (*P*<0.001) and 39% (*P*<0.0001) compared with LPS treatment group, respectively. Intriguingly, tetramethylpyrazine showed higher anti-inflammatory efficacy considering the greater molecular mass. These results showed the anti-inflammatory effects of both candidate compounds in LPS-induced macrophage model and confirmed the feasibility of our *in silico* strategy.

Although the pharmacological effects of active pyrazine compounds in Maotai liquor were both explained by C-T-D network analysis and verified by an *in vitro* experiment, the limitations of our approach still should be acknowledged. The limitations of our approach still should be acknowledged. In this section, the experimental verification was not sufficient to fully prove our prediction, since large-scale experiments performed to validate the pharmacological effects are very time-consuming and labor-intensive, or even ineffective in some circumstances owing to the complexity of biological systems. Thus, more experiments of these prediction results will be necessary to carry out for validating the reliability of our strategy in future studies.

### Pathway analysis

**Figure 4:**
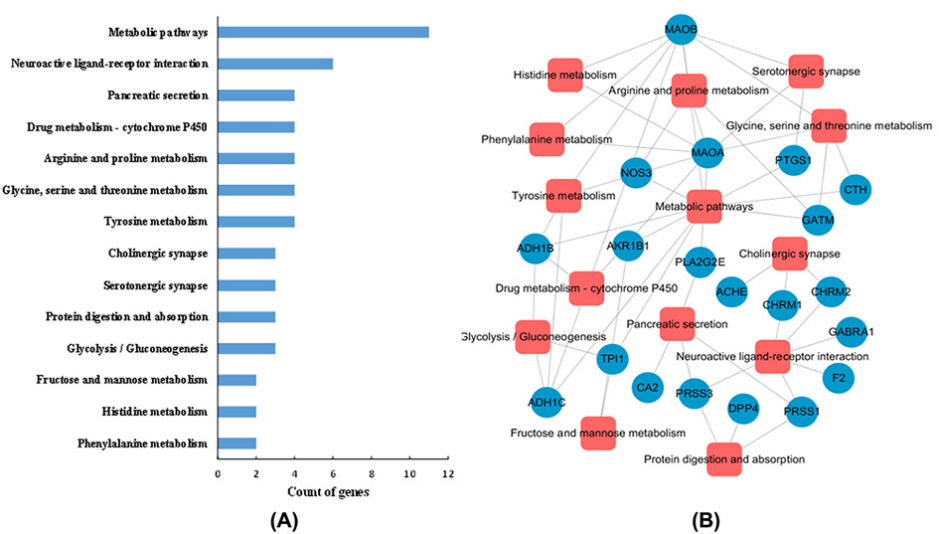
Pathway analysis of the pyrazine compounds in Maotai liquor (**A**) The pathway enrichment analysis of the predicted targets. The *y*-axis indicated the name of enriched pathways related to the targets, and *x*-axis indicated the counts of targets. (**B**) T-P network of candidate targets of active pyrazine compounds in Maotai liquor.

In order to further dissect the potential pharmacological mechanism of pyrazine compounds at the pathway level, all the candidate targets were enriched by DAVID database (https://david.ncifcrf.gov/) to obtain their relevant pathways. As shown in Figure 4, 14 Kyoto Encyclopedia of Genes and Genomes (KEGG) pathways such as metabolic pathways, neuroactive ligand-receptor interaction, and tyrosine metabolism were obtained. The T-P network that consists of targets and their corresponding pathways was visualized in Figure 4B.

Interestingly, the pathways were mainly enriched in three modules, including cardiovascular disease, immune, and inflammation disorders. For instance, 11 target proteins were involved in mediating metabolism pathways which can influence the innate and adaptive immunity through modulating T-cell activation [22]. Tyrosine metabolism (four targets involved) has been demonstrated to be responsible for the inflammatory response since tyrosine kinases are implicated in TLR signaling through inflammatory factor such as tumor necrosis factor, IL-6 and IL-10 [23]. Four targets marked in the drug metabolism-cytochrome P450 which was related to cardiovascular disease. It is suggested that cardiac CYP enzymes can participate in drug metabolism within the heart and influence the survival of cardiomyocytes during ischemic heart disease [24]. These results revealed that pyrazine compounds in Maotai liquor can target multiple proteins that were involved in various pathways, exhibiting their pharmacological effects on the immune and inflammation disorders at the pathway level.

## Conclusions

Maotai liquor is a worldwide consumed distilled alcoholic beverage due to its unique flavor. Pyrazine compounds is one of the most aroma active compounds and has been identified in Maotai liquor. Although analytical chemists have identified many pyrazine compounds from Maotai liquor, its mysteries remain uncovered because of the difficulties in identifying the bioactive substances and their related targets. Moreover, it is still insufficient to systematically and comprehensively interpret the potential pharmacological mechanism of pyrazine compounds in Maotai liquor.

Therefore, in the current study, an integrative approach was provided to explore the bioactive compounds, potential targets, and pharmacological mechanism of pyrazine compounds in Maotai liquor. By incorporating ADME screening, target prediction, pharmacological evaluation, pathway analysis, the active compounds, multiple target proteins, and pathways were obtained. Meanwhile, the pharmacological mechanisms of pyrazine compounds were systematically clarified. Overall, the systematic analysis method established in our study provided a novel strategy to identify candidate compounds and their relevant targets, as well as to explore the potential action mechanisms of pyrazine compounds in Maotai liquor. Moreover, further experiments will be required to validate the results in further research works. Our study will shed lights on the mechanism of pyrazine compounds in Maotai liquor at the system level, allowing for the development of Chinese liquors

## Supplementary Material

Supplementary Table S1

## Abbreviations

ADME: absorption, distribution, metabolism, and excretion
AKR1B1: aldose reductase
BCHE: cholinesterase
CAS: Chemical Abstracts Service
CCK-8: Cell counting KIT-8
C-T-D: compounds-targets-diseases
CYP: Cytochrome P450 proteins
DPP4: dipeptidyl peptidase 4
DTpre: Drug-Target prediction
FBS: Fetal Bovine Serum
IL-6: Interleukin-6
LPS: lipopolysaccharide
OB: oral bioavailability
RF: oral bioavailability
SVM: support vector machine
TLR: Toll-like receptor
T-P: target-pathway
